# Dynamics of a hippocampal neuronal ensemble encoding trace fear memory revealed by *in vivo* Ca^2+^ imaging

**DOI:** 10.1371/journal.pone.0219152

**Published:** 2019-07-03

**Authors:** Liang Zhang, Xuanmao Chen, Carlos Sindreu, Song Lu, Daniel R. Storm, Larry S. Zweifel, Zhengui Xia

**Affiliations:** 1 Department of Environmental and Occupational Health Sciences, University of Washington, Seattle, Washington, United States of America; 2 Department of Pharmacology, University of Washington, Seattle, Washington, United States of America; 3 Department of Psychiatry and Behavioral Sciences, University of Washington, Seattle, Washington, United States of America; University of Wisconsin-Milwaukee, UNITED STATES

## Abstract

Although the biochemical signaling events in area CA1 of the hippocampus underlying memory acquisition, consolidation, retrieval, and extinction have been extensively studied, little is known about the activity dynamics of hippocampal neurons in CA1 during Pavlovian fear conditioning. Here, we use fiber-optic confocal microscopy coupled with the calcium indicator GCaMP6m to monitor neuron activity in freely moving mice during trace fear conditioning. We show that the activity of a group of CA1 neurons increases not only after the stimulus presentations, but also during the stimulus-free trace period when the conditioned mice exhibit a high level of freezing behavior. Therefore, we designate these cells “trace cells”. Interestingly, the activity of the trace cells increases in response to the conditioned stimuli during memory retrieval but diminishes during memory extinction. Importantly, the dynamics of neuron activity exhibit a high degree of correlation with the freezing behavior of the mice, suggesting that a neuronal ensemble responsible for encoding the trace fear memory is repeatedly reactivated during memory retrieval and later extinguished during memory extinction.

## Introduction

Memory is the capacity of an organism to acquire, process, store, and retrieve information based on experience. The prevailing view for the cellular mechanism for memory is that the formation of engrams is due to the formation and strengthening of synaptic connections within populations of neurons [1]. Synaptic plasticity is pivotal to the integration of new information, which requires synchronization of neuronal activity in neuron networks [2]. These populations of neurons or neuronal ensembles, which Donald Hebb called “cell assemblies” [3], implement synaptic plasticity for associative learning when two types of presynaptic inputs trigger activity in the postsynaptic neurons at the same time, thus establishing a temporal relationship between these two types of inputs [4].

Pavlovian fear conditioning is a well-characterized behavioral paradigm for understanding mechanisms of learning and memory, because the behavioral and physiological responses in laboratory animals can be well controlled and examined [5]. During the memory acquisition phase of fear conditioning, a neutral conditioned stimulus (CS), such as a tone, is paired with an aversive unconditioned stimulus (US), such as a mild electric foot shock. This conditioning is subsequently assessed during a test session by exposing the animals to the same CS independently of the US and measuring conditioned responses. After Pavlovian fear conditioning, extensively repeated presentations of the CS without the US lead to extinction of the fear response because the CS no longer predicts the aversive outcome. Memory extinction is an important experimental model to study how learned behaviors diminish in the absence of reinforcement [6].

In delay fear conditioning wherein the CS and the US co-terminate, the amygdala plays a key role [4]. In contrast, in trace fear conditioning a trace period temporally separates the CS and the US and requires distinct neural circuits for associative learning [7, 8]. Namely, trace fear conditioning is a hippocampus-dependent process [9]. Much of the literature on the neurobiology of trace fear conditioning has focused on understanding the mechanisms of hippocampal learning in disease models [10, 11].

Neuron activity causes rapid changes in intracellular free calcium concentrations [12]. Moreover, synaptic plasticity of associative learning during Pavlovian fear conditioning depends on activation of glutamate receptors, including N-methyl-d-aspartate receptors (NMDARs), thereby triggering increases of free calcium in CA1 neurons [4, 7]. Memory retrieval activates the extracellular-regulated kinases (Erk) 1 and 2, key mediators in calcium signaling, in CA1 pyramidal neurons but not in cells of the CA3 or dentate gyrus [13, 14]. Because of the critical role played by calcium in memory formation, calcium imaging technology has been extensively used to track activity of neuronal ensembles [15]. Genetically encoded calcium indicators, such as GCaMPs, are particularly useful in imaging neuronal activity in freely moving mice [16, 17].

Although the molecular and cellular mechanisms of hippocampus-dependent learning have been extensively studied, less is understood about how activity of the CA1 neurons changes during the stimulus-free trace period and whether the changes relate to animal behaviors. In this study, we used the GCaMP6m calcium indicator [16] and fiber-optic confocal (FOC) fluorescence microscopy to visualize activity-dependent calcium signals in hippocampal CA1 neurons in freely moving mice [17]. We identify a group of neurons as “trace cells”; their calcium transients in response to the CS are repeatedly activated during the trace period in training and testing. This activation is diminished by repetitive CS presentations in the absence of US. Neuronal activity during the stimulus-free trace period correlates with the freezing behavior of the mice, suggesting a critical role of the trace period in memory formation, retrieval, and extinction.

## Materials and methods

### Mice

Eight-week-old male C57BL/6 mice were obtained from Charles River (Boston, MA) and allowed to acclimate for two weeks. All animals were housed in standard conditions (12 h light/dark cycle, 22°C) with food and water provided *ad libitum*. The protocol was approved by the Institutional Animal Care and Use Committee of the University of Washington (Protocol Number: 3041–04).

### GCaMP vector

An adeno-associated virus serotype 1 (AAV1) of the GCaMP6m vector (AV-1-PV2823) was purchased from the Penn Vector Core (Philadelphia, PA). This vector expresses GCaMP6m under the human synapsin 1 promoter.

### Stereotaxic surgery and virus injection

All surgery experiments were performed in accordance with the University of Washington regulations on aseptic animal surgery, and all efforts were made to minimize suffering. For all surgical procedures, mice were anesthetized with a mixture of ketamine (130 mg/kg, Zoetis Inc., Kalamazoo, MI) and xylazine (8.8 mg/kg, Akorn Inc., Lake Forest, IL). Animal body temperature was maintained using a heating pad placed underneath the animal. Local anesthetic (2% lidocaine, Hospira Inc., San Clemente, CA) was applied to all incisions.

For viral injections and probe insertion, a small hole was drilled in the skull directly above the dorsal hippocampal CA1 region. The following stereotaxic coordinates relative to Bregma were used: A-P: 1.95 mm × a (a = lambda-bregma distance/4.2), M-L: 1.35 mm. A cannula (Mauna Kea Technologies, Paris, France) was attached to the skull with dental acrylic (Lang Dental Manufacturing Co. Inc., Wheeling, IL) and small set screws. One week after the surgery, 0.5 μl AAV1-GCaMP6m virus was injected through the cannula into the CA1 (D-V: 1.55 mm). Mice recovered and expressed GCaMP6m for eight weeks prior to the behavior experimentation and imaging. Mice were single-housed after surgery.

### Trace fear conditioning and imaging

Data was acquired using a CellVizio 488 imaging system coupled with a NeuroPak fiber-optic confocal fluorescence microscope (Mauna Kea Technologies, Paris, France). On experimental days, mice were anesthetized with 2% isoflurane and positioned on the stereotaxic frame. The fiber-optic (a 300 μm diameter fiber-bundle of 8517 fibers, axial resolution: 15 μm; lateral resolution: 3.3 μm) was lowered through the cannula into CA1 (D-V: 1.55 ± 0.05 mm) where fluorescence signal was detected and locked into place. Only light conveyed and collected by the same microfiber reaches the photodetector, thereby rejecting diffusion and out-of-focus light. Mice were placed in the behavior room for 30 minutes and then in the conditioning chamber for 10 minutes prior to experiments. Mice were conditioned in a modified, sound-attenuated chamber (Med-Associates, Fairfax, VT). The trace fear conditioning paradigm is adapted from published method with minor modifications [18]. The training session consists of 7 trials, 65 seconds per trial with an inter-trial interval of 195 seconds. Each trial consists of a 10-second baseline period, a 15-second presentation of tone (80 dB, 3000 Hz), a 30-second trace period followed by a 1-second electric foot shock (0.7 mA), and a 9-second post-shock period. Memory retrieval and extinction was tested in a different context. The interior surface of the shock chamber, including all walls and the grid floor, was covered by a white plastic corrugated board three hours after the training session. The board was sprayed with 1% acetic acid. The retrieval/extinction session consists of 4 tests with 5 trials each. Each trial is 47 seconds, consisting of a 10-second baseline period and a 5-second presentation of tone followed by a 30-second trace period and a 2-second post-trace period. The inter-trial interval is 123 seconds. The purpose of using a shortened duration of tone in test sessions is to avoid fear memory being extinguished too quickly and to capture a relatively gradual change in the animal response to the tone. The four tests were separated by 1-hour intervals. Mouse behavior in the entire training session and four test sessions was recorded on a digital camera from above and fluorescent signals were recorded simultaneously at 11.7 Hz through the fiber-optic microscope.

### Data processing

Freezing behavior is defined as cessation of movement with four paws on the ground and no head or body movement besides breathing. It was manually scored by an experimenter and the percentage of freezing was calculated for each 10-second bin. Fluorescent data was analyzed offline using ImageCell software (Mauna Kea Technologies, Paris, France) and statistical programming language R (version 3.5.1). Fluorescent regions of interest (ROI) for individual cells were manually circled with a diameter of ~10–20 μm and minimal detection by four individual adjacent fibers. ROIs were drawn based on fluorescence during the baseline period of the first trial in training and then were applied to all subsequent trials. The total number of cells recorded from each animal varied from 10 cells to 22 cells. The intensity signals of ROIs for all trials were acquired and imported into R for further analysis.

Fluorescence decay was corrected with a one-phase decay model with data from the first 10 seconds and last 2 seconds for each cell and each trial. Fitted curves were subtracted from the raw data to generate normalized data. Change in fluorescence (ΔF) was calculated by subtracting the average normalized fluorescence during baseline imaging (10 seconds) from each time point during the imaging session (65 seconds for training, 47 seconds for tests). Percent change in fluorescence (%ΔF/F) was calculated by dividing the ΔF by the average asymptote (F) from the decay curve. Data was smoothed using a 5-point sliding average. %ΔF/F was then converted to z-scores by normalization to the baseline signal of each cell and the baseline mean of the population.

### Experimental design and statistical analysis

All means are reported as means ± standard error. Repeated-measures designs were analyzed using the mixed-effects restricted maximum likelihood (REML) model. Tukey’s test was used for *post hoc* pairwise multiple comparisons. Comparison of neuron population activities between early trials (1&2) and late trials (6&7) in training for each time point was made using student’s t-test with Benjamini-Hochberg correction.

To classify trace cells, we first took a conservative approach, using three standard deviations from the mean z-score of baseline activities during the first 10 seconds of training trials 1&2 and trials 6&7 as the threshold to calculate the activation rate (fraction of time when cell activity was above the threshold) at baseline or during the trace period. A cell was classified as a trace cell if its trace period activation rate was higher than ten-fold of its baseline activation rate, an arbitrary but conservative criterion. To determine if a trace cell is reactivated or inhibited in the four tests, we used three standard deviations from the mean z-score of baseline activities during the first 10 seconds of all trials in all four tests as the threshold to calculate the baseline activation or inhibition rate. A cell was scored as activated if its activation rate during the trace period was higher than ten-fold of its baseline activation rate. Similarly, a cell was considered inhibited if its inhibition rate during the trace period was ten-fold or higher of its baseline inhibition rate.

To study the correlation between mouse freezing behavior and neuron activity in the trace period, we calculated a freezing discrimination index and a neuron activity discrimination index to summarize the differences between training (trials 6&7) and each test session for each mouse. Instead of using just freezing percentages, we used the freezing discrimination index to account for variability in individual behavior. The freezing discrimination index, ranging from -1 to 1, was calculated to represent the differences in freezing between training (trials 6&7) and each test for each mouse: freezing discrimination index = (F_Training_−F_Test_)/ (F_Training_ + F_Test_), where F is the percentage of freezing time. A freezing discrimination index of 1 means that the mouse froze during training but did not freeze during test, -1 means the mouse froze during test but did not freeze during training and 0 means no difference between training and test.

To represent the differences in neuron activities between training (trials 6&7) and each test for each mouse, we calculated a neuron activity discrimination index based on the area under receiver operating characteristic curve (auROC) [19], a commonly used method in signal detection theory: neuron activity discrimination index = (auROC– 0.5) × 2. This index also ranges from -1 to 1. Thus, a neuron activity discrimination index approaching 1 means higher neuron activity in training compared to test, an index approaching -1 means lower neuron activity in training compared to test and 0 means equal neuron activity. Pearson’s correlation tests were used to establish a relationship between two variables.

### Immunohistochemistry

Mice were euthanized with carbon dioxide after the completion of imaging. Brain tissues were fixed with 4% paraformaldehyde (Cat. P6148, Sigma-Aldrich, St. Louis, Missouri) in phosphate-buffered saline (PBS, pH 7.4) overnight and dehydrated with 30% (w/v) sucrose (Cat. 8360–06, Avantor Performance Materials, Center Valley, Pennsylvania) in PBS solution at 4°C for 48 hours. Coronal brain sections (30-μm-thick) were then subjected to immunohistochemistry staining using primary antibodies against GFP (1:500, Cat. A-11120, Invitrogen, Carlsbad, California). Nuclei were stained with Hoechst 33342 (2.5 μg/ml, Invitrogen).

## Results

### Memory retrieval and extinction in trace fear conditioning

**Fig 1:**
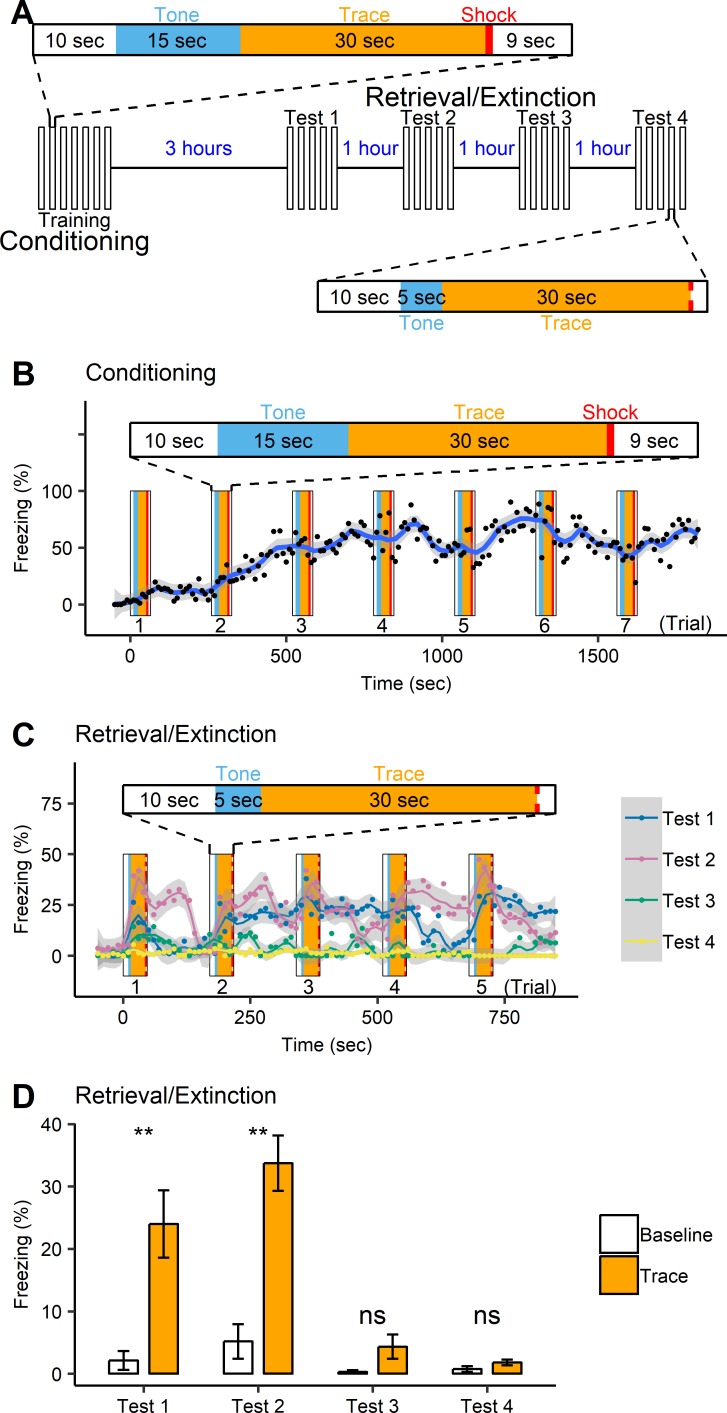
Freezing behavior of C57B1/6 mice (n = 5) during trace fear conditioning with multiple test sessions. (A) A schematic diagram of one training session and four test sessions in trace fear conditioning. The dashed line in a test trial indicates the time of an expected shock. (B) During training, mice exhibited rapid acquisition of freezing behavior. The whole training session consists of 7 trials, 65 seconds/trial with a 15-second tone and a 1-second mild foot shock. Each dot represents the percentage of freezing time averaged across all mice in a 10-second bin. (C) Freezing behavior in response to the tone only in a novel context gradually diminished during multiple test sessions. Each test consists of 5 trials, 47 seconds/trial with a 5-second tone. The dashed line in the diagram indicates the time of an expected shock. Each dot represents the percentage of freezing time averaged across all mice in a 10-second bin. Each smooth line represents a moving average curve for a test. Grey area indicates 95% confidence interval. (D) Mean freezing of 5 trials in each test session, comparing the baseline and the trace period. ** p < 0.01; ns, not significant (p > 0.05).

To investigate the activities of neuronal ensembles in the hippocampus during memory acquisition, retrieval, and extinction, the fiber-optic imaging probe has to be tightly locked in position for continuous imaging. To accomplish this, we designed a trace fear conditioning paradigm in which fear memory can be retrieved and eliminated within a timeframe of one day (Fig 1A). This approach avoids the need to remove and reinsert the fiber-optic probe and ensures that the same group of cells are continuously imaged at their exact locations over the course of the experiment.

The trace fear conditioning consisted of one training (conditioning) session (7 trials) and four test (retrieval/extinction) sessions (5 trials each). The percentage of freezing was measured in 10-second bins in each session. During training, the average freezing level increased gradually in the first four trials and then plateaued around 70% for the remaining three trials (Fig 1B), suggesting that the CS-US pairs were able to trigger and maintain responses to the aversive stimuli. Three hours later mice were placed in a novel context for four tests of tone-elicited fear responses, separated by one-hour intervals. There were main effects of Test and Time as well as the effect of the Test × Time interaction (Fig 1C, REML: Test, F_(3, 1452)_ = 2.69, p = 0.045; Time, F_(90, 1452)_ = 1.67, p = 0.0001; Test × Time, F_(270, 1452)_ = 1.40, p = 0.0001). Freezing during the baseline period (30 seconds before the first tone) was compared to the mean freezing during the trace period (Fig 1D). There was a significant main effect of Period (Baseline, Trace) and a significant interaction of Period × Test (REML: Period, F_(1, 108)_ = 10.47, p = 0.0016; Period × Test, F_(3, 108)_ = 6.20, p = 0.0006). Mice displayed sharply increased freezing behavior in response to the tone in test 1 and test 2, but not in test 3 and test 4 (Tukey, Baseline vs. Trace: test 1, p = 0.0016; test 2, p < 0.0001; test 3, p = 0.55; test 4, p = 0.88). In test 3 and test 4, the percentage of freezing during the trace period was significantly reduced relative to tests 1 and 2 (Tukey: test 1 vs. test 2, p = 0.59; test 1 vs. test 3, p = 0.058; test 1 vs. test 4, p = 0.024; test 2 vs. test 3, p = 0.0013; test 2 vs. test 4, p = 0.0004; test 3 vs. test 4, p = 0.99). Mice displayed very little freezing behavior in test 4, indicating memory extinction. These results showed that repeated CS-US presentations were able to trigger CS-associated fear response and continuing CS presentations without the US induced fear memory extinction in our experimental paradigm.

### Neuron activity is significantly increased during conditioning

**Fig 2:**
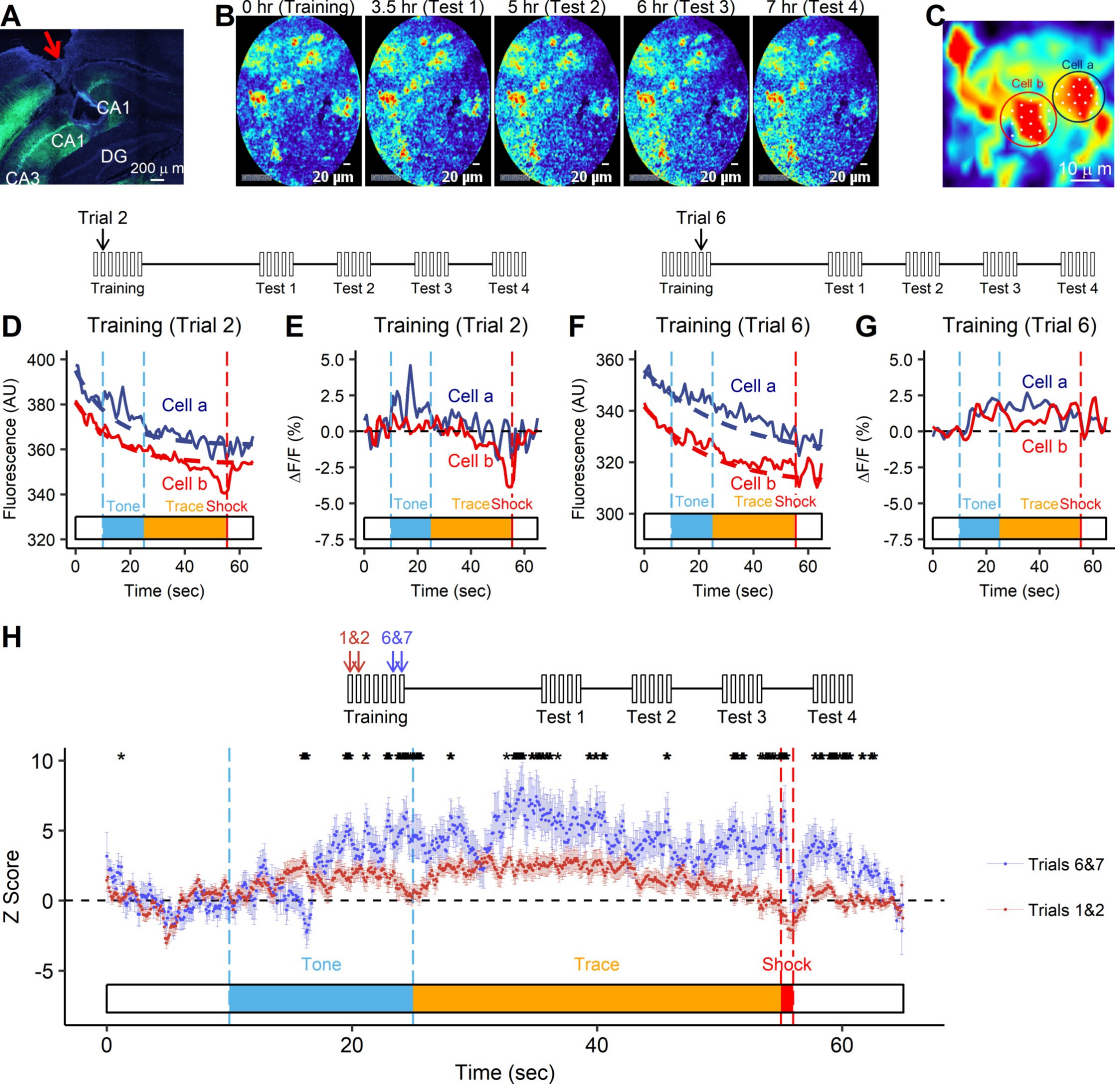
Characterization of calcium fluorescent signals in CA1 neurons during the training session. (A) Immunohistochemistry showing the expression of GCaMP6m (green) in the hippocampal CA1. Nuclei were stained with Hoechst, shown in blue. The red arrow indicates the probe insertion site. The injury site (hole) on the CA1 was made when manually taking the imaging probe out after imaging was completed. (B) Representative images of GCaMP6m fluorescent signals recorded by the fiber-optic *in vivo* in all sessions, showing stable signals over the course of the experiments. (C) A representative image of two ROIs. White dots indicate the locations of the centers of individual fibers. Fibers outside the circles are not shown. (D, F) Fluorescent signals (AU, arbitrary unit) acquired from the two cells circled in panel C in an early trial (D, trial 2) and a late trial (F, trial 6) of training. Colors correspond to the colors of circles in panel C. The dashed lines under the fluorescence signals indicate fitted one-phase decay curves. (E, G) Percentage change in fluorescence of the two cells circled in panel C. Colors correspond to the colors of circles in panel C. (H) Population means of z-scores of the first two trials (trials 1&2, red) and the last two trials (trials 6&7, blue) of training. Asterisks indicate the time points when the two means of trials 1&2 and trials 6&7 are significantly different (p < 0.05, two-tailed paired t-test with Benjamini-Hochberg corrections).

We investigated the activity of the neuronal ensembles associated with trace fear conditioning in freely moving mice via changes of intracellular Ca^2+^. We injected the Ca^2+^ indicator AAV1-syn-GCaMP6m into the dorsal hippocampal CA1 of C57BL/6 mice through a cannula attached to the skull. The expression of the GCaMP6m protein in the CA1 was confirmed by immunofluorescent staining with anti-GFP antibodies (Fig 2A). Before the trace fear conditioning experiment for each mouse, the fiber-optic was inserted into CA1 through the cannula. The probe was tightly secured to the cannula to avoid image shifting during animal moving. Fluorescent signals from neurons were recorded following the trace fear conditioning paradigm. The signals were stable during the 7-hour-long experiment with multiple imaging sessions (Fig 2B), enabling us to capture signals from the same cells across all sessions (Fig 2C). In total, we analyzed signals from 80 cells in five mice. During training, cells were imaged for a total of 65 seconds for each trial. To determine whether the fluorescent signals changed in response to the CS or US, we first fitted each curve to a one-phase decay model (Fig 2D and 2F) and calculated %ΔF/F (Fig 2E and 2G). Representative cells showed increased signal peaks for longer periods in a late trial (Trial 6) compared to that in an early trial (Trial 2) (Fig 2E and 2G).

We plotted the population means of the fluorescent signals during the early trials (Trials 1&2) and the late trials (Trials 6&7) of training (Fig 2H). To statistically compare the early trials and the late trials, we aggregated data points into 1-second bins. There were significant main effects of Phase (early trials, late trials) and Time, as well as a significant interaction of Phase × Time (REML: Phase, F_(1, 10191)_ = 8.80, p = 0.003; Time, F_(64, 10191)_ = 9.51, p < 0.0001; Phase × Time, F_(64, 10191)_ = 3.61, p < 0.0001). At many time points, the cell population means of the late trials were significantly higher than those of the early trials during CS presentations as well as after US presentation. Interestingly, the population means of the late trials were well above those of the early trials throughout the entire trace period, showing several peaks. These data suggest that as the animals were conditioned to form tone-associated fear memory, the activity of the neuron population in CA1 was simultaneously evoked by the stimulations of the CS-US pair.

### Identification of the trace cells

**Fig 3:**
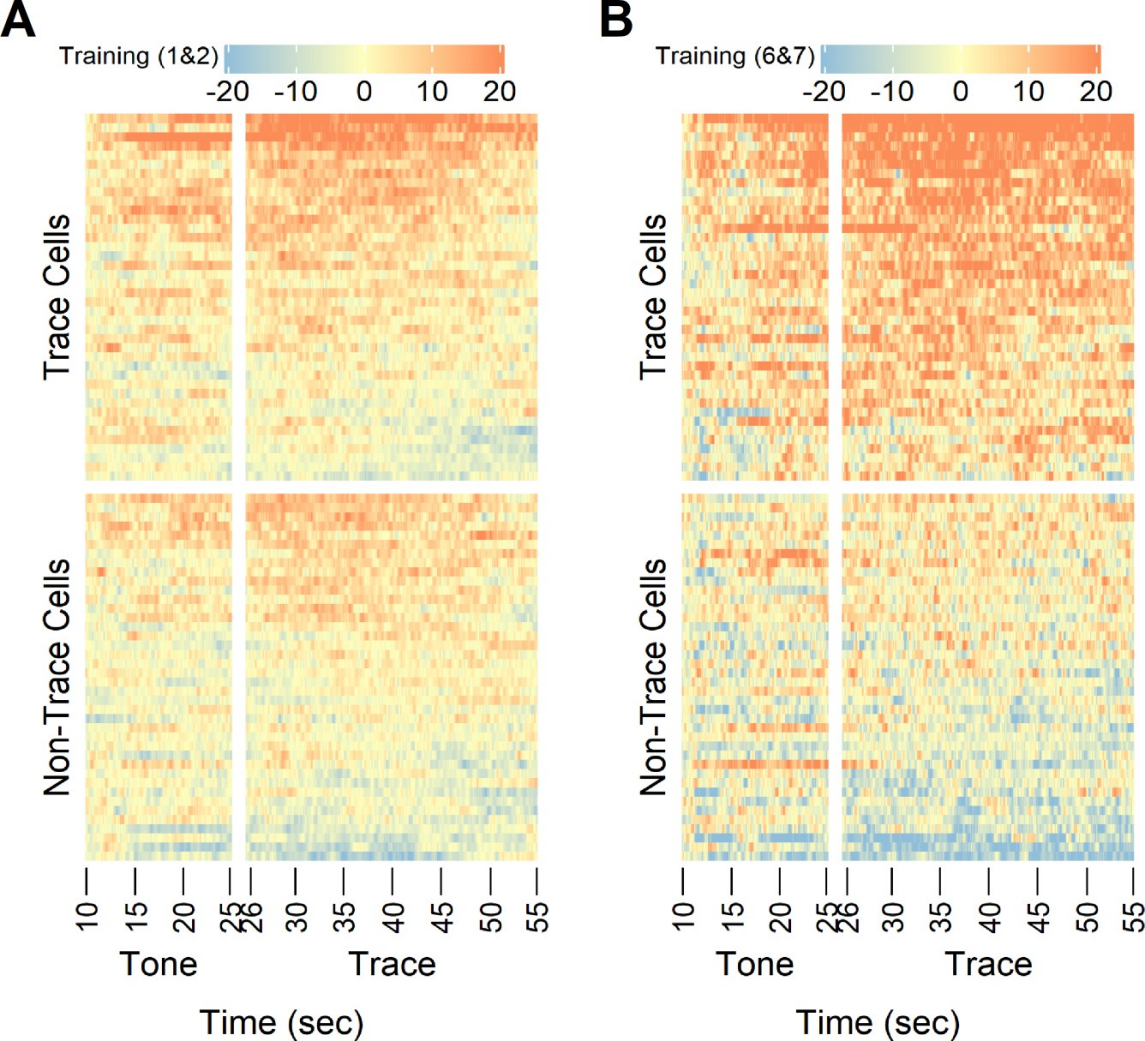
**Heat maps of z-scores of all cells during the first two trials (A, trials 1&2) and the last two trials (B, trials 6&7) of training.** The trace cells were determined based on data of the trace period in trials 6&7 (See Materials and methods). Cells were ranked from highest average z-score of the trace period to the lowest.

In order to identify cells belonging to a neuronal ensemble associated with the fear memory, we ranked the average z-scores in the trace period for all individual cells and plotted heat maps comparing activity signals during the tone and trace of trials 1&2 (Fig 3A) and trials 6&7 (Fig 3B). Many cells showed largely elevated activity in the late trials compared to that in the early trials while others did not change. To quantitatively separate these two populations of cells, we calculated the activation rate in the 10-second baseline period of the late trials and used ten-fold baseline activation rate as the threshold to determine cells that were active during the trace period. We identified 40 out of 80 cells which showed an activation rate above this threshold and defined them as “trace cells”. Consistent with the population activity in Fig 2D, many trace cells were activated during the tone and through the entire trace period, suggesting that these trace cells may be critical to forming the neuronal ensemble for encoding the fear memory.

### Activity of the trace cells during memory retrieval and memory extinction

**Fig 4:**
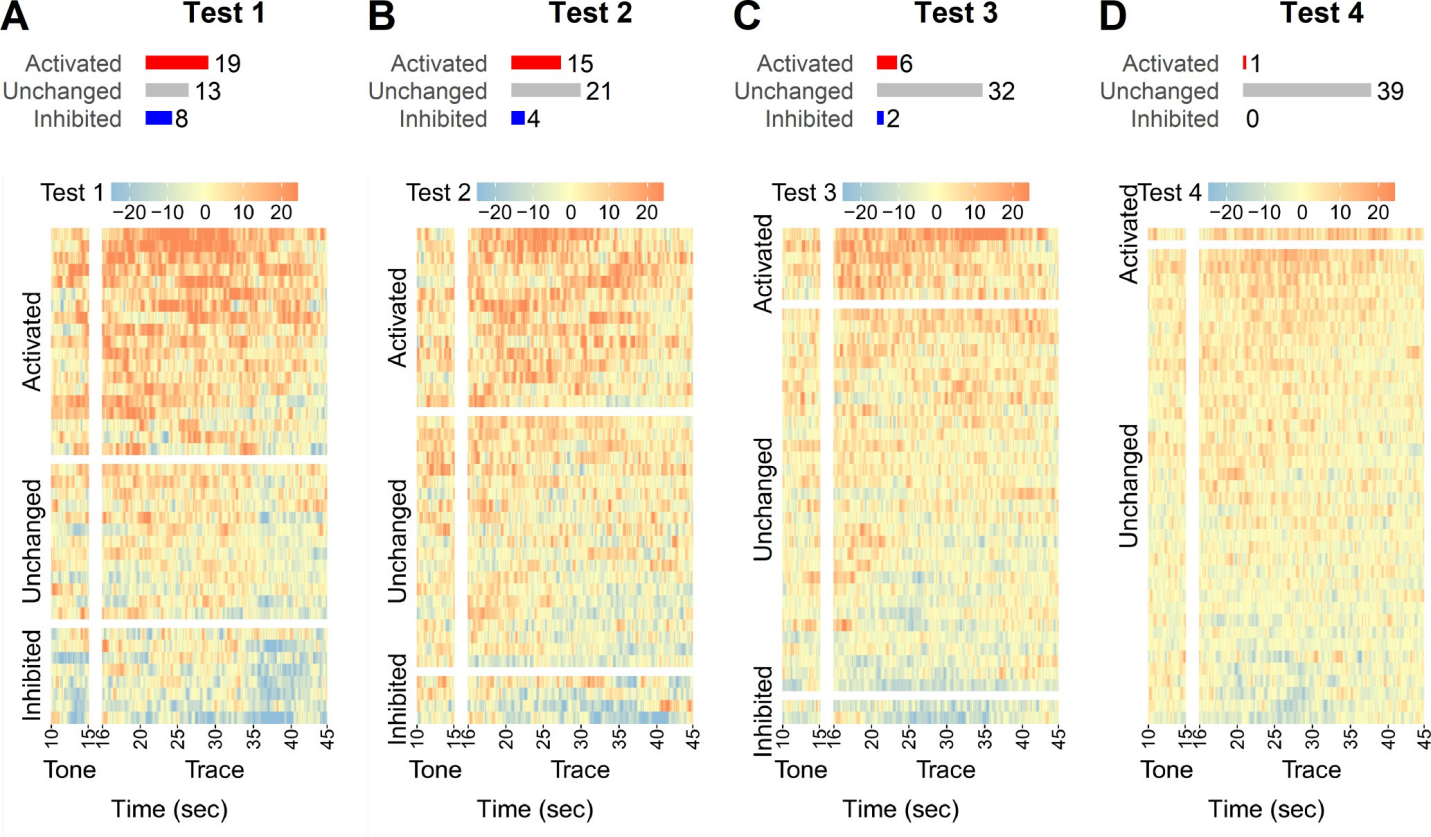
**Heat maps of z-scores of the trace cells in four tests during the retrieval/extinction session: test 1 (A), test 2 (B), test 3 (C) and test 4 (D).** Cells were divided into three categories (activated, unchanged, inhibited) based on data of the trace period in each test respectively. Cells were ranked from highest average z-score of the trace period to the lowest in each test.

To test whether memory retrieval can reactivate trace cells, we investigated how the trace cells identified during training responded to the CS in the absence of the US during memory retrieval. Three hours after the training, mice were put in a novel context for four tests of tone-elicited fear. In test 1 (Fig 4A) and test 2 (Fig 4B), 19 and 15 of the previously identified 40 trace cells were reactivated respectively during the trace period, suggesting that memory retrieval partially reactivated the neuronal ensemble of trace cells. Interestingly, in test 3 (Fig 4C), when the freezing behavior of the mice was largely diminished (Fig 1C), only 6 of 40 trace cells were reactivated. In test 4 (Fig 4D), only one trace cell was reactivated, suggesting that activity of the trace cells was extinguished in concert with the extinction of the freezing behavior.

**Fig 5:**
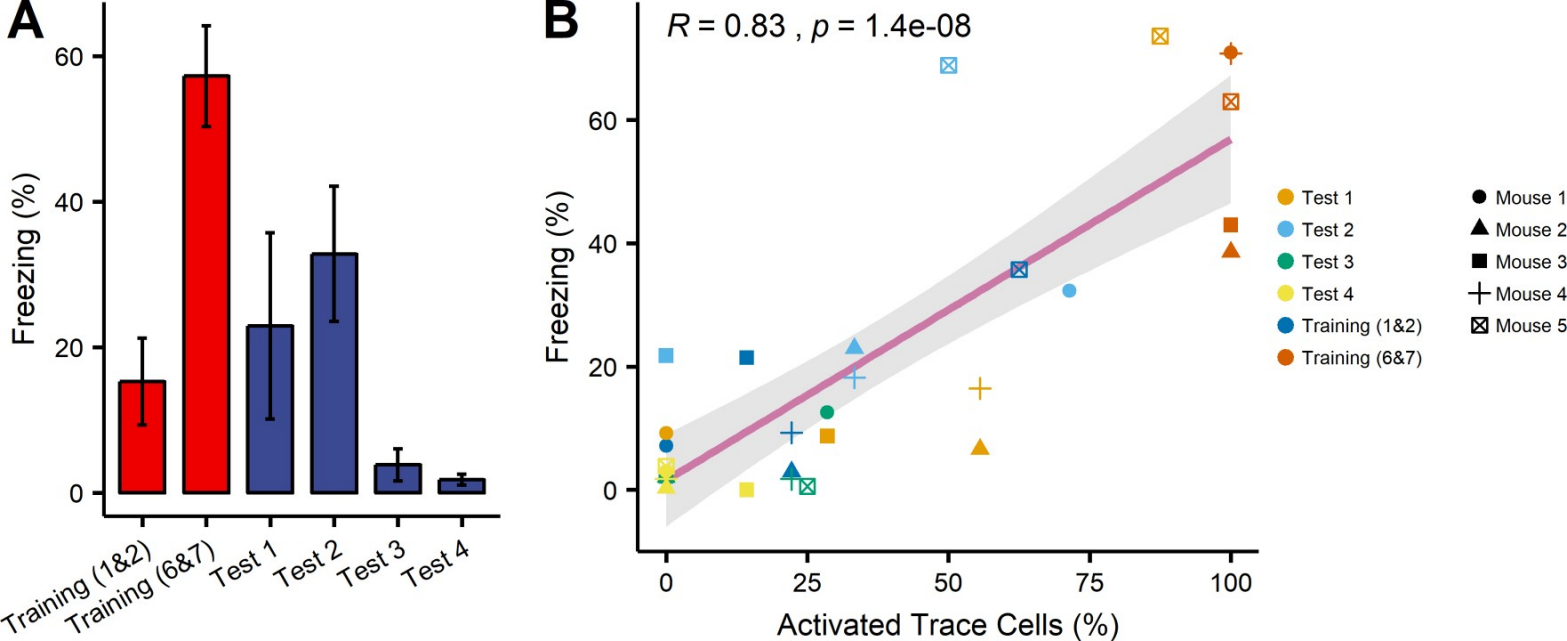
Correlation between percentage of activated trace cells and percentage of freezing. (A) Average percentage of freezing during the CS and the trace interval in training (red, trials 1&2, trials 6&7) and four tests (blue). (B) Percentages of activated trace cells and percentages of freezing for individual mice were positively correlated (R = 0.83, p = 1.4×10^−8^). Data was fit to a linear regression model (magenta line). Grey area indicates 95% confidence interval.

The prominent changes in activity signals of trace cells during memory retrieval and extinction prompted us to examine how well the freezing behavior and neuron activity were correlated. We calculated the percentage of total freezing time during the CS and the trace interval in trials 1&2 in training, trials 6&7 in training, and each of the four tests (Fig 5A). The percentage of freezing increased from 15% in trials 1&2 to 55% in trials 6&7 during training. Mice exhibited tone-elicited fear response in test 1 and test 2 and lost fear response in test 3 and test 4.

We then plotted the percentage of freezing time during the CS and the trace interval versus the percentage of activated trace cells for individual mice (Fig 5B). The data was fitted to a linear regression model to examine the correlation. We observed a highly correlated (R = 0.83, p = 1.4×10^−8^) relationship. Taken together, these data suggest that the percentage of activated trace cells may reflect tone-elicited fear memory of the mice.

**Fig 6:**
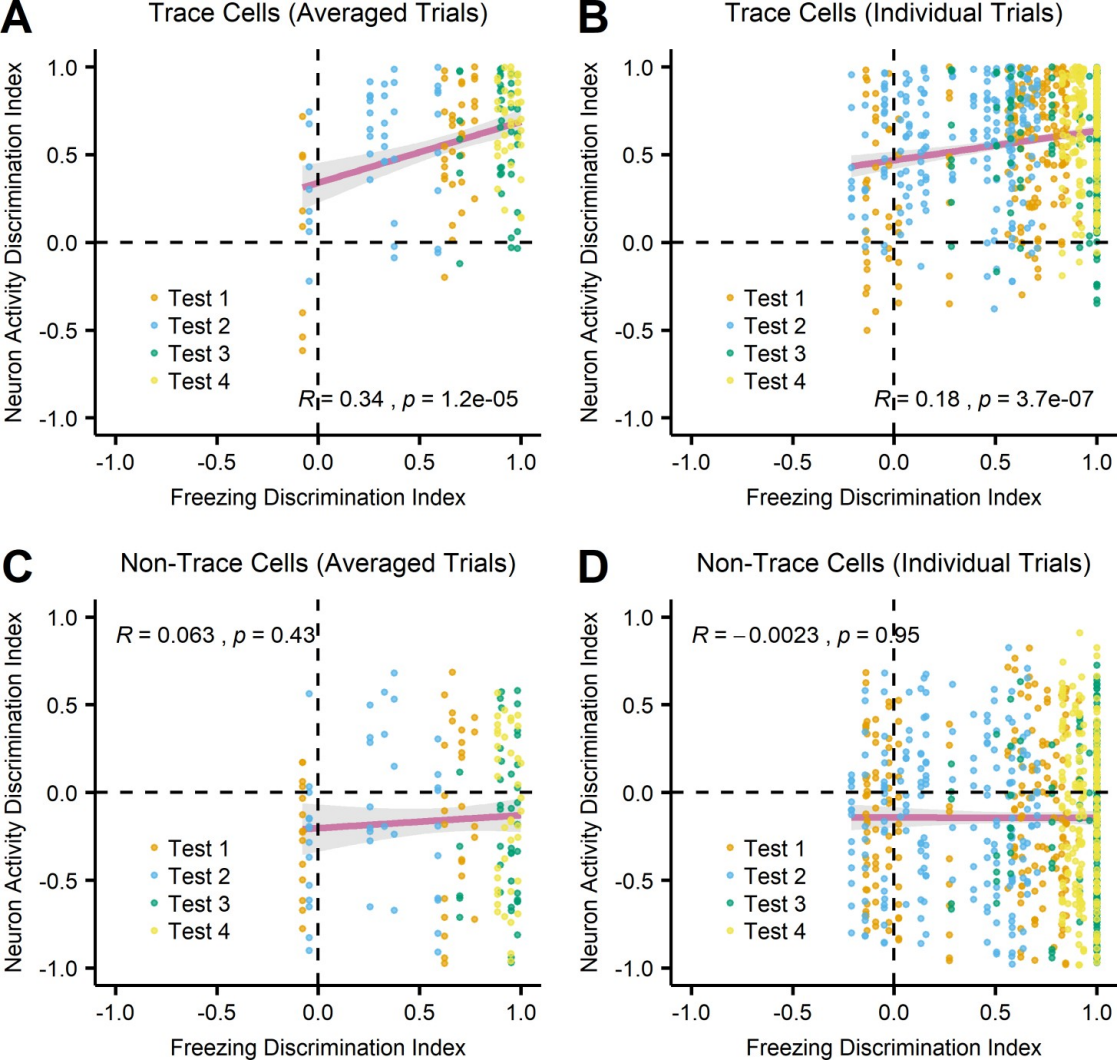
Correlation between freezing behavior and neuron activity. (A, C) Freezing discrimination index and neuron activity discrimination index for individual cells were calculated based on data of the trace period in training (trials 6&7) and in each test averaged across five trials. For the trace cells (A), there was a positive correlation between freezing discrimination and neuron activity discrimination (R = 0.34, p = 1.2×10^−5^). For the non-trace cells (C), freezing discrimination index and neuron activity discrimination index of averaged trials were not correlated (R = 0.063, p = 0.43). (B, D) Freezing discrimination index and neuron activity discrimination index for individual cells were calculated based on data of the trace period in training (trials 6&7) and in each test for each individual trial. There was a positive correlation between freezing discrimination and neuron activity discrimination for trace cells (B, R = 0.18, p = 3.7×10^−7^), but not for the non-trace cells (D, R = -0.0023, p = 0.95). Data was fit to a linear regression model (magenta line) in each panel. Grey area in each panel indicates 95% confidence interval.

To further examine the correlative relationship between freezing behavior and neuron activity, we analyzed the data for each trial of four tests in all individual mice. To quantify the differences of neuron activities during the trace period between training (trials 6&7) and each test for each cell, we calculated a neuron activity discrimination index based on the area under the receiver operating characteristic (auROC) curve. The auROC values were rescaled (index = (auROC– 0.5) × 2) with a range of -1 to 1 so that an index approaching 1 indicates higher neuron activity in training compared to test, an index approaching -1 indicates lower neuron activity in training compared to test, and 0 indicates equal neuron activity between training and test. We then calculated a freezing discrimination index to indicate the differences in freezing behavior during the trace period between training (trials 6&7) and each test. A freezing discrimination index of 1 means that the mouse froze during training but did not freeze during test, -1 means the mouse did not freeze during training but froze during test, and 0 means no difference between training and test. We plotted the neuron activity discrimination index as a function of the freezing discrimination index. This analysis allows us to test whether changes in neuron activity of individual cells in individual mice reflect changes in freezing behavior and compare the trace cells to the non-trace cells. Calculated with either averaged trials (Fig 6A) or individual trials (Fig 6B), there was a positive correlation between freezing discrimination and neuron activity discrimination for the trace cells (averaged trials: R = 0.34, p = 1.2×10^−5^; individual trials: R = 0.18, p = 3.7×10^−7^). For the non-trace cells, however, there was no correlation between freezing discrimination and neuron activity discrimination of either averaged trials (Fig 6C, R = 0.063, p = 0.43) or individual trials (Fig 6D, R = -0.0023, p = 0.95). Taken together, there is a clear distinction between the trace cells and the non-trace cells: only the trace cells showed a strong correlation between their neuron activity and the freezing behavior of mice.

## Discussion

In this study, we used an *in vivo* calcium imaging technique to monitor CA1 neuron activity in freely moving mice during and following trace fear conditioning. We identified a neuronal ensemble of trace cells in the hippocampal CA1 that was activated during fear memory formation in trace fear conditioning. These trace cells were partially reactivated during memory retrieval and extinguished during memory extinction. An examination of the relationship between the behavioral response of conditioned fear memory and neuron activity revealed a positive correlation between freezing behavior with the activity of the trace cells, but not with the non-trace cells.

During trace fear conditioning, animals exhibit weaker behavioral responses compared to the classic delay fear conditioning, possibly because the memory trace decays as a function of time, resulting in weaker associative learning [20]. Another possible explanation is that inhibitory learning occurs as the animals learn that the presence of the CS indicates the absence of the US during trace fear conditioning [7, 21]. Interestingly, several studies demonstrated that behavioral responses peak around the time of the expected US presentations [7, 22]. This suggests a unique excitatory learning mechanism which enables the animals to not only establish CS-US associations but also acquire the timing of responding. A study in humans also supports the idea that temporal conditioning is one important aspect of Pavlovian conditioning [23]. Correspondingly, previous studies have shown that hippocampal CA1 neurons in conditioned animals become active at the expected time of the shock in the trace retention test, suggesting a possible link between behavioral responses and neuron activity [24, 25]. All of these proposed mechanisms indicate that the trace period between the CS and the US presentations is critical for memory formation in trace fear conditioning. In the current study, we observed that in the last two training trials, the Ca^2+^ signals of neurons in the hippocampal CA1 increased during the tone and after the foot-shock (Fig 2H), suggesting responsiveness to the stimuli. Interestingly, compared to the first two training trials, the signals in the last two training trials were elevated in several time windows during the stimulus-free trace period, supporting the notion that the trace period is central to establishing the CS-US linkage.

In contrast to data presented here, previous studies of electrophysiological activity of hippocampal CA1 neurons during trace fear conditioning suggested that there was no clear sign of neuron activity in the trace interval [24, 26]. However, data for mean changes of activity in CA1 neurons, rather than the activities of each single neurons, were presented in those reports, masking the possibility that a subset of the recorded neurons could be activated during the trace interval. Our identification of trace cells that are active during the trace period is consistent with more recent studies using large-scale ensemble recording or two-photon calcium imaging [25, 27, 28]. These studies on trace fear conditioning or trace eyeblink conditioning have shown that in anticipation of the US, a group of CA1 neurons are active during the trace period. However, it is not clear if the same population of CA1 neurons can be reactivated across multiple memory retrieval sessions and how these neurons change during memory extinction. We report here that trace cells, identified during training, are activated during memory retrieval but this activation diminishes as memory decays.

Studies of contextual fear conditioning in animals and humans suggest that engrams of contextual representations may be composed of widely distributed networks of neuronal ensembles located in the hippocampus, the medial prefrontal cortex (mPFC), and the amygdala [5]. The neural circuits for fear conditioning are best understood in delay fear conditioning, which involves the hippocampus encoding context representations and projecting to the amygdala directly or indirectly via the mPFC. The hippocampus and the mPFC play distinct temporal roles in encoding trace fear memory, revealed by the respective necessity of the hippocampus in short-term memory and the mPFC in long-term memory, but not vice versa [29]. These pathways converge in the amygdala which serves multiple roles in establishing direct associations between contexts and stimuli [2]. Inactivation of the amygdala in various ways disrupts trace fear conditioning, suggesting a critical role of amygdala in the neuronal circuit [30–32]. However, there seems to be numerous distinctions in neural circuits between trace fear conditioning and delay fear conditioning [33]. Several studies illustrate that the disruption of the dorsal hippocampus affect trace conditioning, but not delay conditioning, suggesting the importance of a selective role of the hippocampus in the neural circuits of trace fear conditioning [31, 34, 35]. By tracking activity of individual cells throughout all training trials we identified a group of cells in the CA1 and named them “trace cells”, because they showed elevated activity during the trace period in the last two trials of training (Fig 3). These trace cells may be part of a neuronal ensemble in the hippocampal CA1 responsible for encoding fear memory, thus suggesting a model that CA1 neurons serve a key role in trace-mediated memory acquisition and consolidation.

The Hebbian theory suggests that experience constantly reshapes the brain by modifying neuronal connections within a neuronal network, providing a simple explanation for synaptic plasticity, whereby the formation of an engram from a specific experience results from repeated and persistent stimulation of the simultaneously activated neurons [4]. This increase of synaptic strength in the neuronal ensembles increases the likelihood of the same spatiotemporal pattern being retrieved and recreated electrophysiologically and being expressed behaviorally (e.g. freezing) by interactions with retrieval cues [1]. In the US-independent tests we observed that the trace cells identified in training were at least partially reactivated by the CS, possibly because the cue was able to trigger the learning-induced synchronization among the trace cells (Fig 4A and 4B).

The current theoretical views of memory extinction fall into two major categories: associative loss and new inhibitory learning. In the model of associative loss, extinction is a process of unlearning or deconditioning, in which the original CS-US associative memory is erased, at least partially. The model of new inhibitory learning suggests that extinction is a new form of learning. A CS-no US association develops to interfere with the original memory. Thus, conditioned response diminishes due to the inhibitory relationship between the CS-US relationship and the CS-no US relationship [6]. In the last two tests we performed, we observed that very few trace cells were reactivated by the CS. Rather, after repetitive presentations of the CS independently of the US, all but one trace cells seemed to become quiet, neither activated nor inhibited (Fig 4C and 4D). This observation is consistent with the associative loss model. However, we cannot simply rule out the model of new inhibitory learning because we were only able to survey a small set of cells in the CA1 region due to technical limitations. Indeed, some statistical learning models suggest that these two mechanisms may not be mutually exclusive. These newer models subsume both previous models and suggest a balance between contributions from updating the original fear memory and the formation of new extinction memory [36, 37].

Although the calcium imaging technique used in this study is limited to a subset of neurons comprising an engram, it has provided unique opportunities to visualize neuron dynamics during memory acquisition, retrieval, and extinction, and to distinguish between the trace cells and the non-trace cells. With the recorded activity dynamics of individual cells, we were able to connect cellular data and behavioral data by examining the relationship between neuron activities and freezing behavior (Fig 5). There is a strong positive relationship between the extent of trace cell activity and the extent of fear response. Furthermore, ROC analysis allows us to investigate how activity dynamics of individual cells correspond to changes of fear response in individual mice (Fig 6). The distinction observed between the trace cells and the non-trace cells further strengthens the notion that the trace cells belong to a neuronal ensemble in the hippocampal CA1 recruited for encoding the fear memory.

In summary, data in this study identify a group of CA1 cells whose intracellular Ca^2+^ levels increase during the trace period of training. These cells can be reactivated during memory retrieval but not when memory extinction occurred. These trace cells may be key players in encoding trace fear memory.
